# Supplementary material for: Morphological and molecular convergences in mammalian phylogenetics
Source: Nat Commun. 2016 Sep 2;7:12758. doi: 10.1038/ncomms12758 (PMC5025827; doi:10.1038/ncomms12758)
Supplement: Supplementary Information — Supplementary Figures 1 - 8 and Supplementary Tables 1 - 3 [file ncomms12758-s1.pdf]

Supplementary Figures

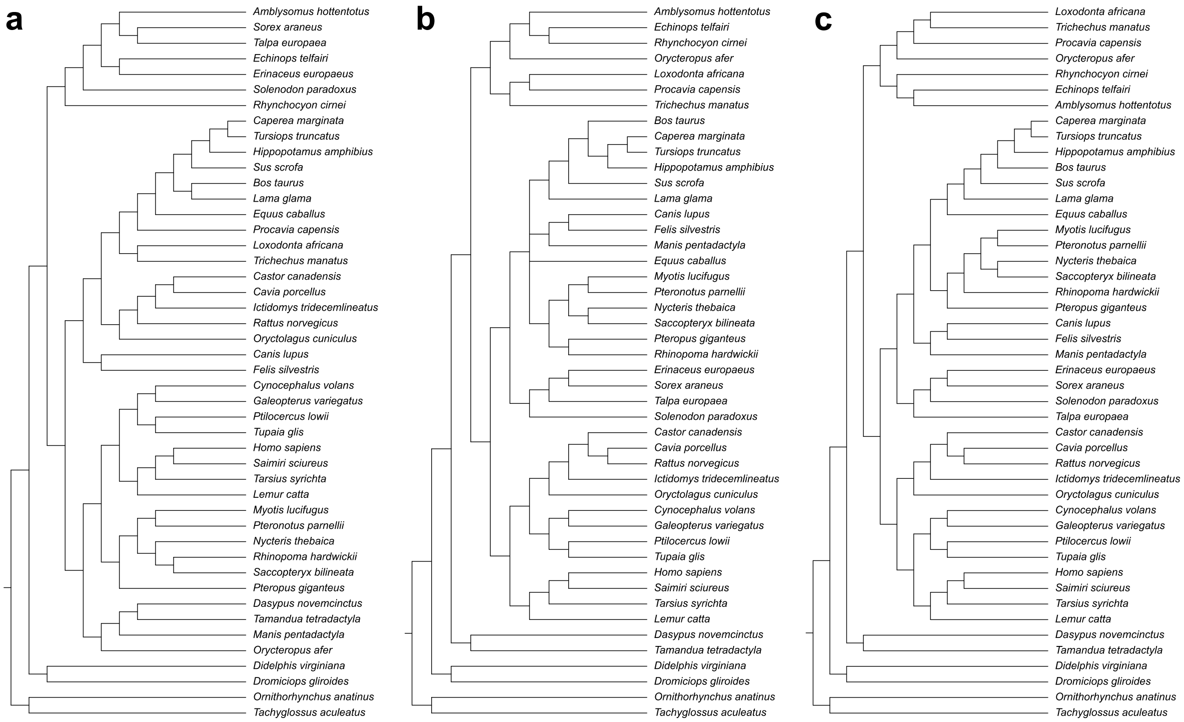

Supplementary Figure 1. Tree topologies used in the analysis of convergence. (**a**) The parsimony tree based on all morphological characters of all 86 species. Only the 46 extant species are shown here. (**b**) The parsimony tree based on all molecular characters of the 46 extant species. (**c**) The parsimony tree based on all morphological and molecular characters of all 86 species. Only the 46 extant species are shown here.

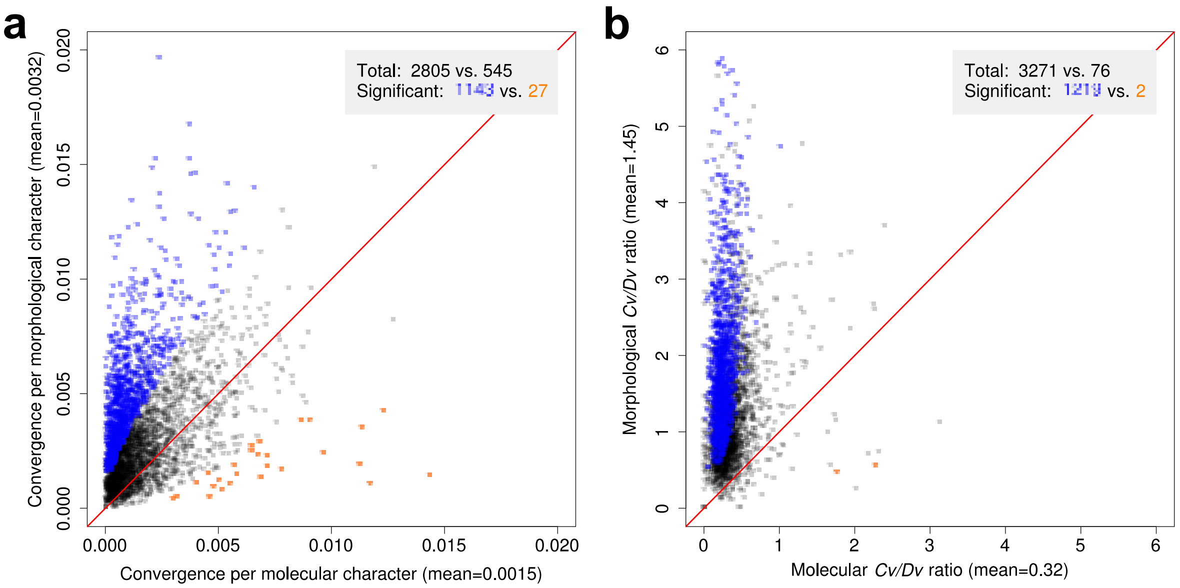

Supplementary Figure 2. Whole-tree analysis of convergence based on the total evidence tree. (**a**) Mean number of convergences per morphological character and that per molecular character for each branch pair examined. (**b**) Convergence/divergence (*Cv*/*Dv*) ratio for each branch pair. In (a) and (b), each dot represents a branch pair. In the grey box of each panel, 'total' refers to the numbers of dots above and below the diagonal, respectively, and 'significant' refers to the numbers of dots significantly (at *Q*-value of 0.05) above (blue) and below (orange) the diagonal, respectively (dots on the diagonal are not counted). Total number of dots above the diagonal significantly exceeds that below the diagonal in both panels ($P < 1\times10^{-4}$, bootstrap test).

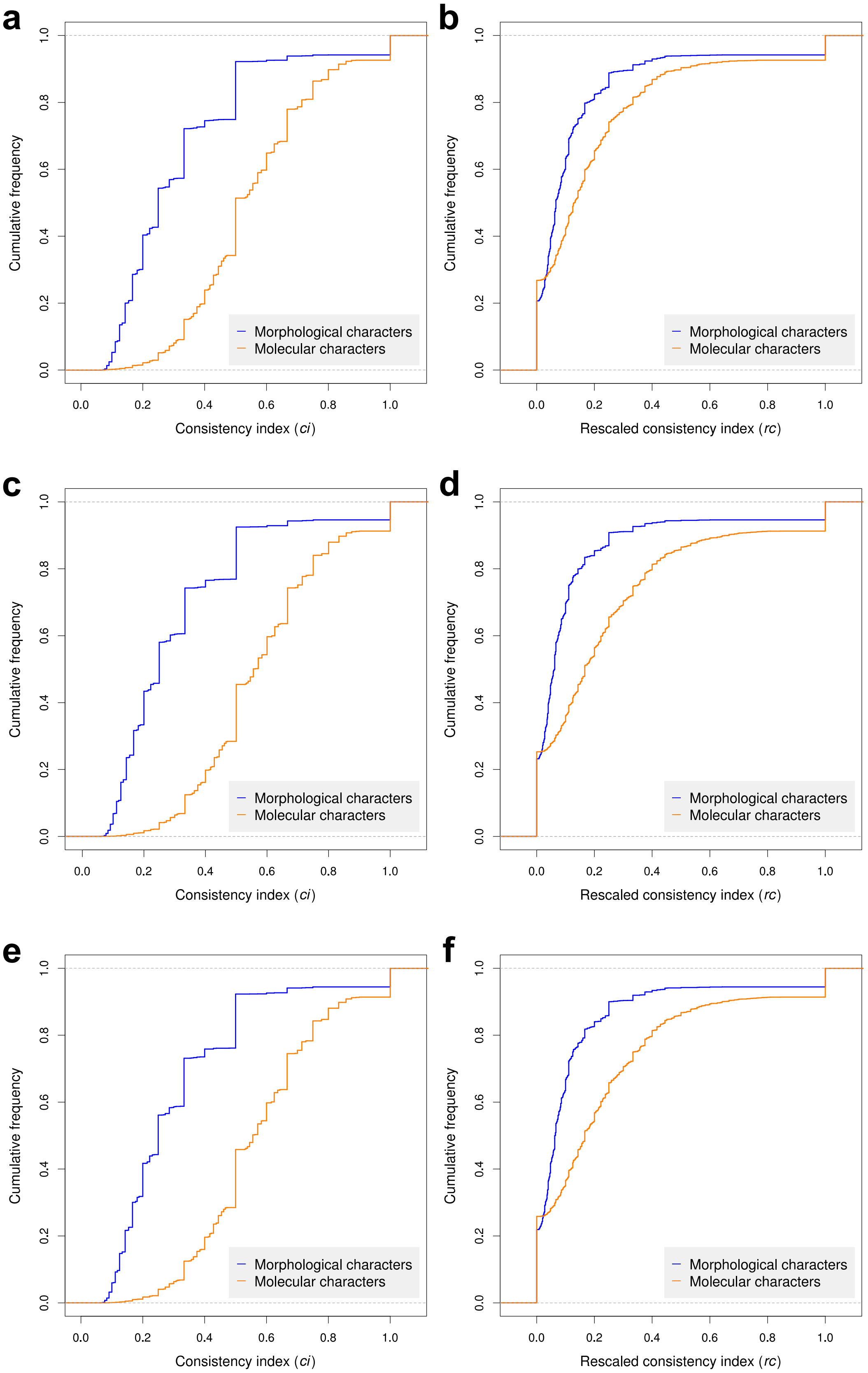
a
b
c
d
e
f
Cumulative frequency
0.0
0.2
0.4
0.6
0.8
1.0
Consistency index (ci)
Rescaled consistency index (rc)
Morphological characters
Molecular characters

Supplementary Figure 3. Consistency index and rescaled consistency index are generally higher for molecular characters than morphological characters. (**a**) Cumulative frequency distributions of consistency index for parsimony-informative morphological characters and molecular characters based on the morphological tree. The difference between the two distributions is significant ($P < 1\times10^{-300}$, Mann-Whitney U test). (**b**) Cumulative frequency distributions of rescaled consistency index for parsimony-informative morphological characters and molecular characters based on the morphological tree. The difference between the two distributions is significant ($P < 3\times10^{-47}$, Mann-Whitney U test). (**c**) Cumulative frequency distributions of consistency index for parsimony-informative morphological characters and molecular characters based on the molecular tree. The difference between the two distributions is significant ($P < 1\times10^{-300}$, Mann-Whitney U test). (**d**) Cumulative frequency distributions of rescaled consistency index for parsimony-informative morphological characters and molecular characters based on the molecular tree. The difference between the two distributions is significant ($P < 2\times10^{-137}$, Mann-Whitney U test). (**e**) Cumulative frequency distributions of consistency index for parsimony-informative morphological characters and molecular characters based on the total evidence tree. The difference between the two distributions is significant ($P < 1\times10^{-300}$, Mann-Whitney U test). (**f**) Cumulative frequency distributions of rescaled consistency index for parsimony-informative morphological characters and molecular characters based on the total evidence tree. The difference between the two distributions is significant ($P < 4\times10^{-111}$, Mann-Whitney U test).

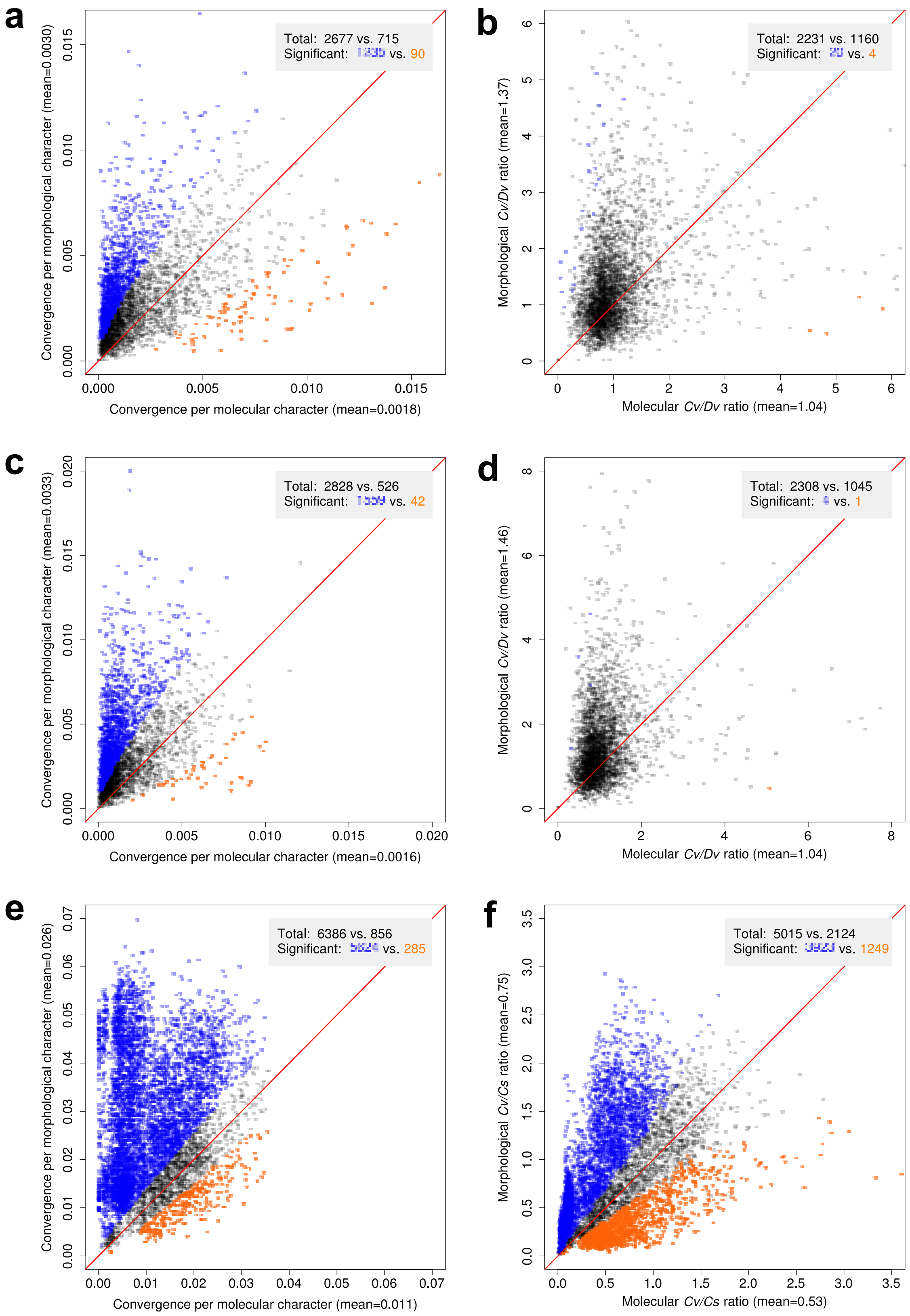
a
Total: 2677 vs. 715
Significant: vs. 90
Convergence per morphological character (mean=0.0030)
Convergence per molecular character (mean=0.0018)
b
Total: 2231 vs. 1160
Significant: vs. 4
Morphological Cv/Dv ratio (mean=1.37)
Molecular Cv/Dv ratio (mean=1.04)
c
Total: 2828 vs. 526
Significant: vs. 42
Convergence per morphological character (mean=0.0033)
Convergence per molecular character (mean=0.0016)
d
Total: 2308 vs. 1045
Significant: vs. 1
Morphological Cv/Dv ratio (mean=1.46)
Molecular Cv/Dv ratio (mean=1.04)
e
Total: 6386 vs. 856
Significant: vs. 285
Convergence per morphological character (mean=0.026)
Convergence per molecular character (mean=0.011)
f
Total: 5015 vs. 2124
Significant: vs. 1249
Morphological Cv/Cs ratio (mean=0.75)
Molecular Cv/Cs ratio (mean=0.53)

Supplementary Figure 4. Whole-tree analysis (**a**-**d**) and quartet analysis (**e**,**f**) with nucleotide sites being the molecular data. (**a**) Mean number of convergences per morphological character and that per molecular character for each branch pair examined under the morphological tree. (**b**) Convergence/divergence (*Cv*/*Dv*) ratio for each branch pair under the morphological tree. (**c**) Mean number of convergences per morphological character and that per molecular character for each branch pair examined under the molecular tree. (**d**) *Cv*/*Dv* ratio for each branch pair under the molecular tree. Labels, legends, and color schemes in (a)-(d) follow Fig. 1. (**e**) Mean number of convergences per morphological character and that per molecular character for each quartet examined. (**f**) Convergence/consistency (*Cv*/*Cs*) ratio for each quartet. Labels, legends, and color schemes in (e) and (f) follow Fig. 2.

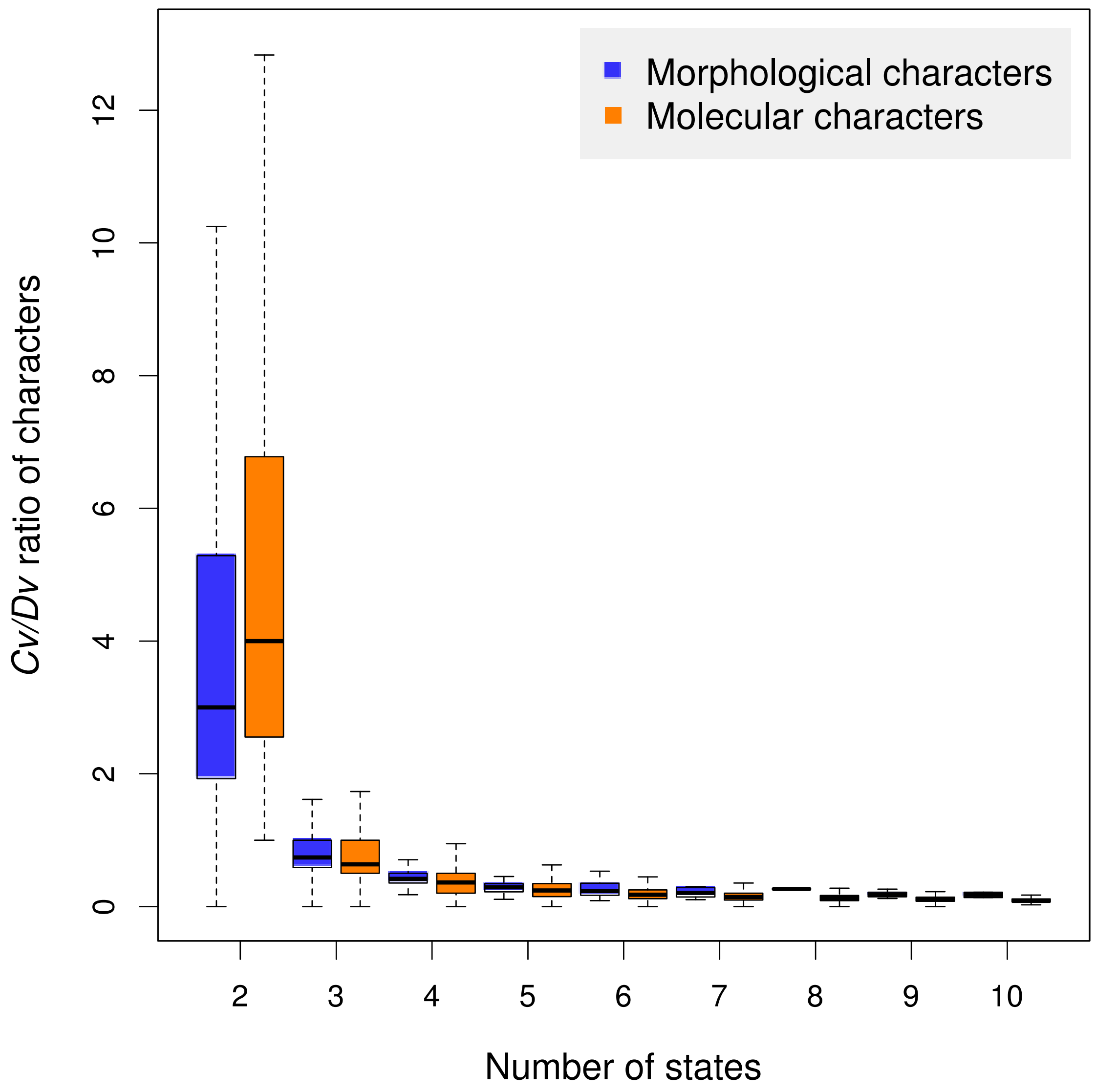

Supplementary Figure 5. *Cv/Dv* ratio decreases as the number of states increases. The *Cv/Dv* ratio of a character is the sum of convergences across all branch pairs divided by that of divergences. *Cv/Dv* ratios are calculated under the molecular tree.

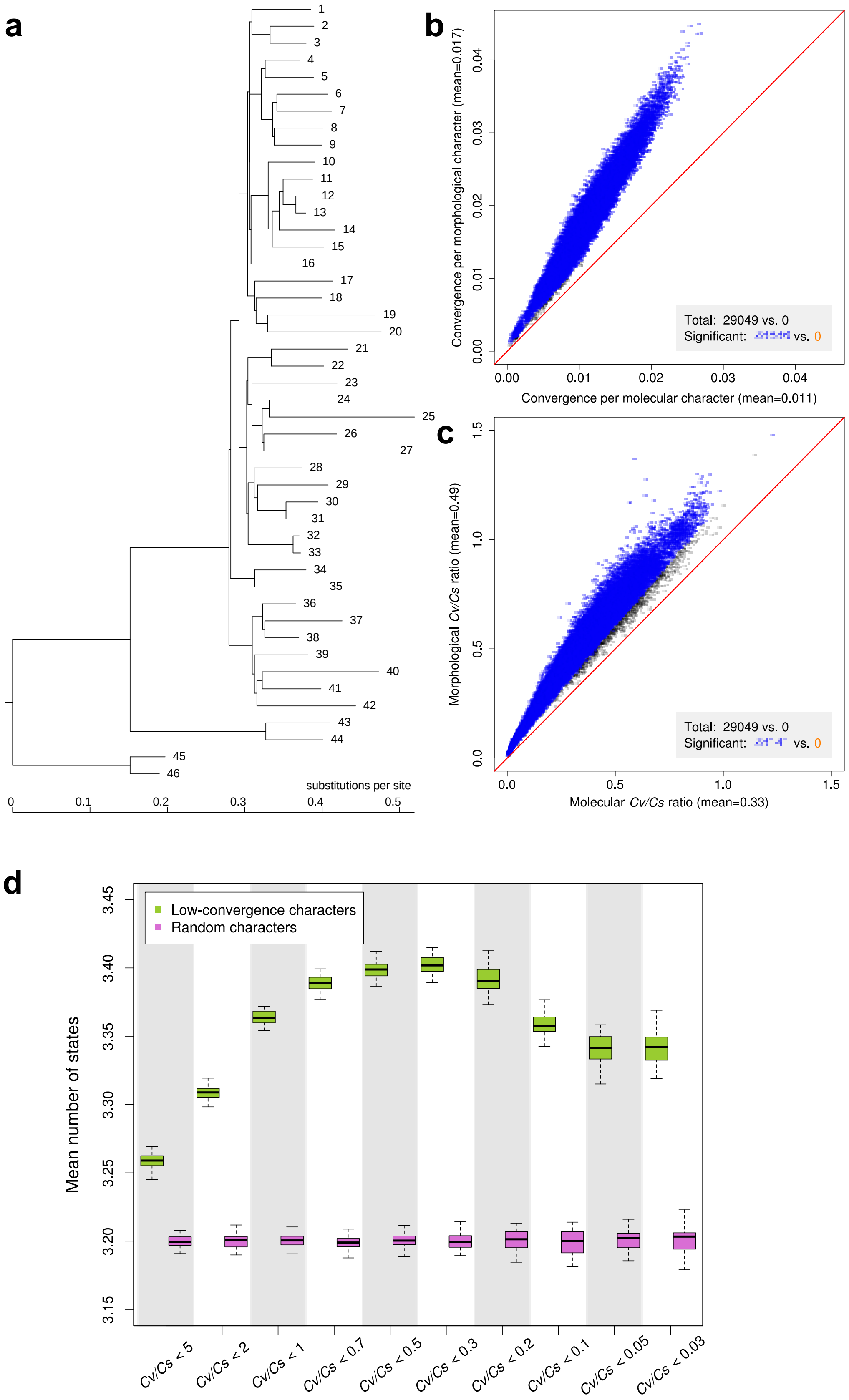
a
substitutions per site
b
Convergence per morphological character (mean=0.017)
Convergence per molecular character (mean=0.011)
Total: 29049 vs. 0
Significant:
vs. 0
c
Morphological Cv/Cs ratio (mean=0.49)
Molecular Cv/Cs ratio (mean=0.33)
Total: 29049 vs. 0
Significant:
vs. 0
d
Mean number of states
Low-convergence characters
Random characters
Cv/Cs < 5
Cv/Cs < 2
Cv/Cs < 1
Cv/Cs < 0.7
Cv/Cs < 0.5
Cv/Cs < 0.3
Cv/Cs < 0.2
Cv/Cs < 0.1
Cv/Cs < 0.05
Cv/Cs < 0.03

Supplementary Figure 6. Properties of simulated morphological and molecular characters. (**a**) Tree used in the simulation, which is the nucleotide maximum likelihood tree from the original study. (**b**) Convergence per character for all quartets that show the same phylogenetic relationships in the morphological and molecular trees. (**c**) *Cv*/*Cs* ratios for the same quartets. Each dot represents a quartet. Annotations and legends follow Fig. 2b and 2c. In (b) and (c), each dot represents a quartet. Convergence and consistency information was obtained from quartet analysis. Numbers of states and steps were directly recorded in the simulation. The data were from the first of the 50 simulations. Number of dots above the diagonal significantly exceeds that below the diagonal ($P < 1\times10^{-4}$, bootstrap test). (**d**) The remaining characters after the removal of high-convergence characters tend to have larger numbers of states, compared with those of the same numbers of randomly picked characters from the original data. The top and bottom edges of a box respectively represent the first and third quartiles of the distribution from 50 simulations, while the thick line inside the box represents the median. The two whiskers show the maximum value not greater than the 1st quartile plus 1.5 times the box height and the minimum value not smaller than the 3rd quartile minus 1.5 times the box height, respectively. Differences between all pairs of boxes are significant ($P < 1\times10^{-8}$) by Mann-Whitney U test.

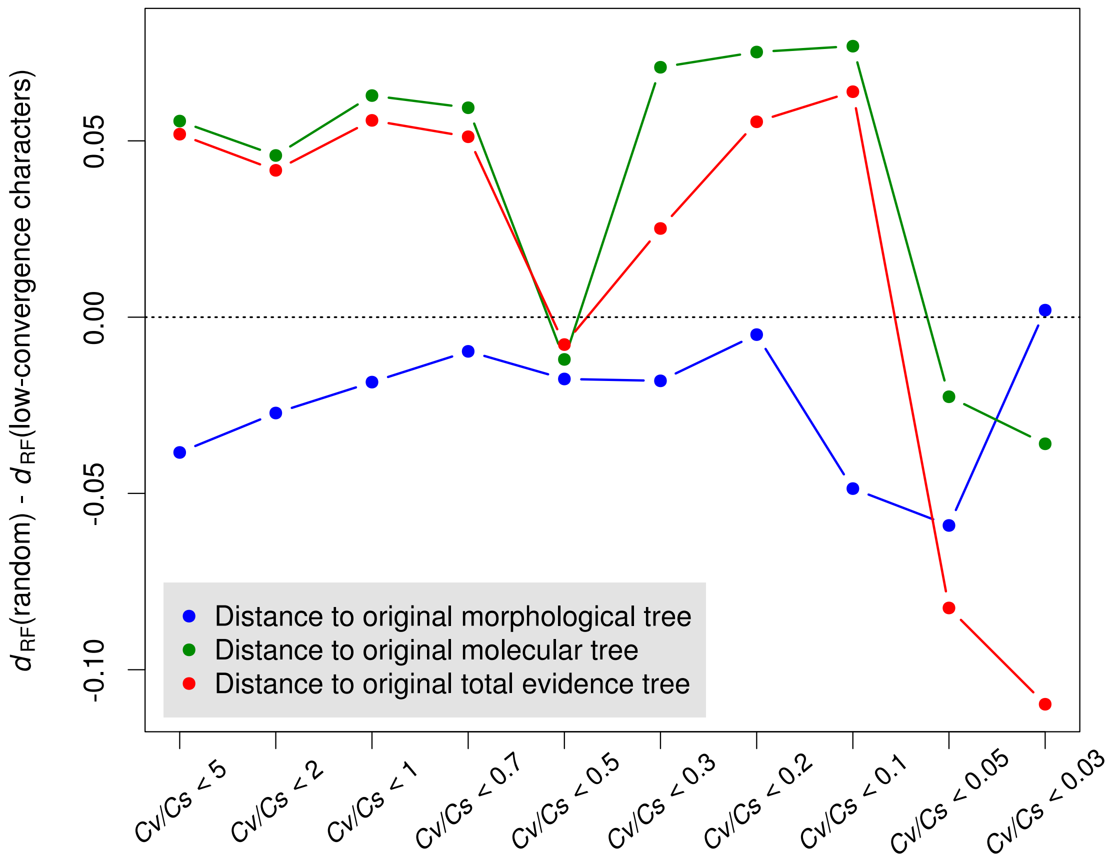

Supplementary Figure 7. Decrease in Robinson-Foulds distance (d_{RF}) between the inferred tree and an original tree after the removal of high *Cv*/*Cs* characters, relative to that after the removal of the same number of randomly picked characters. Positive Y-axis values show that, relative to removing random characters, removing high *Cv*/*Cs* characters makes the tree closer to the original tree being compared.

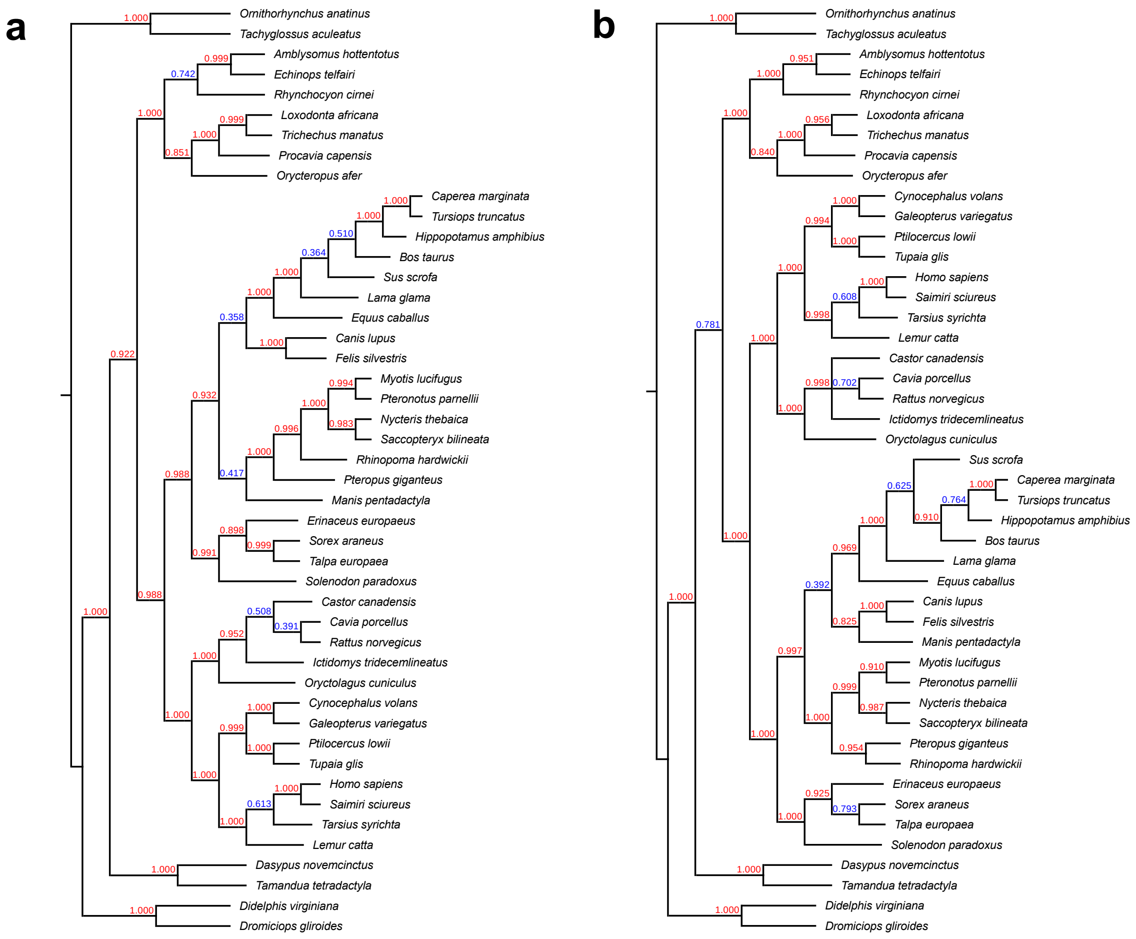

Supplementary Figure 8. Parsimony trees of mammals before and after the removal of high-convergence characters. (**a**) Parsimony tree of 46 extant species based on all informative morphological and amino acid characters. (**b**) Parsimony tree of 46 extant species based on informative morphological and amino acid characters with *Cv/Cs* < 0.2. Branch labels indicate the proportions of 1000 bootstrapped trees that support the subdivision of 46 species by this branch. Bootstrap values lower than 0.8 are colored in blue. All 86 species are included in the phylogenetic and bootstrap analyses, although only extant species are shown here.

Supplementary Tables

Supplementary Table 1. Convergence level negatively correlates with number of states after the control of evolutionary rate in the actual data

Spearman's rank correlation	Partial correlation with number of states (controlling for the number of steps)		
	all informative characters	morphological characters	molecular characters
Cv/Dv ratio of characters	ρ = -0.86, P < 1E-300	ρ = -0.79, P < 1E-300	ρ = -0.77, P < 1E-300
Cv/Cs ratio of characters	ρ = -0.31, P = 2E-202	ρ = -0.19, P = 1E-27	ρ = -0.22, P = 8E-63

Amino acid sites are used as molecular data. Evolutionary rates (number of steps) are inferred on the basis of the morphological tree.

Supplementary Table 2. Convergence level negatively correlates with number of states after the control of evolutionary rate in the actual data

Spearman's rank correlation	Partial correlation with number of states (controlling for the number of steps)		
	all informative characters	morphological characters	molecular characters
Cv/Dv ratio of characters	ρ = -0.78, P < 1E-300	ρ = -0.78, P < 1E-300	ρ = -0.76, P < 1E-300
Cv/Cs ratio of characters	ρ = -0.18, P = 3E-155	ρ = -0.19, P = 1E-28	ρ = -0.17, P = 2E-124

Nucleotide sites are used as molecular data. Evolutionary rates (number of steps) are inferred on the basis of the morphological tree.

Supplementary Table 3. Convergence level negatively correlates with number of states after the control of evolutionary rate in the simulated data

Spearman's rank correlation	Partial correlation with number of states (controlling for the number of steps)		
	all informative characters	morphological characters	molecular characters
Cv/Cs ratio of characters	ρ = -0.32, P < 1E-300	ρ = -0.14, P = 5E-79	ρ = -0.33, P < 1E-300

Simulated amino acid sites are used as molecular data. Evolutionary rates (number of steps) are recorded during the simulation.
